# The signaling role of feedback in the repeated public goods game: Experimental evidence from the laboratory

**DOI:** 10.1371/journal.pone.0299196

**Published:** 2024-02-29

**Authors:** Chi-Hsiang Liu, Shih-Feng Tsai, Ting-Cih Chen, Hsiu-Wen Cheng

**Affiliations:** 1 School of Economic and Trade, Hubei University of Economics, Wuhan, China; 2 Department of Economics and Finance, Ming Chuan University, Taoyuan City, Taiwan; 3 Taiwan Youth Employment Demonstration Site of Hubei University of Economics, Hubei University of Economics, Wuhan, China; Teesside University, UNITED KINGDOM

## Abstract

This paper empirically examines the signaling role of feedback in the repeated public goods game. To eliminate the potential impact of feedback’s informative function, we test whether the provision of detailed yet redundant feedback leads to increased contributions. Our findings demonstrate that redundant information significantly promotes contributions. Given the equal informative power in both treatments, the observed increase in contributions can be attributed to the signaling effect. Furthermore, an examination of cooperative disposition heterogeneity reveals that conditional cooperators primarily utilize feedback for its informative function, while free riders primarily exploit it for its signaling function. These results offer empirical evidence of the signaling function of feedback and offer valuable insights into the design of feedback provision in repeated public goods settings.

## Introduction

The public goods game (PGG) simply and effectively models the phenomenon of conflict between individual and collective benefit [1], making it a commonly used basis for experimental design in research on social dilemma problems [2]. Facilitating cooperation is crucial to managing social dilemmas and promoting collective benefits in human society. Previously, experimental economists dedicated significant efforts to examining mechanisms aimed at stabilizing contribution behavior over time. Please see [3] for an extensive survey of laboratory experiments investigating factors that affect contribution levels. In a repeated PGG setting, feedback information disclosed at the end of each period, which reveals the actions of players within the group during that period, crucially affects the following contribution decisions. The use of information can take two directions. On the one hand, players can utilize feedback information to develop their beliefs about the contributions of others in subsequent periods. This understanding subsequently impacts their own decisions related to contributions [4]. This informational function has been empirically verified in prior studies [5,6]. On the other hand, players can use feedback information to signal their willingness to cooperate in the future [7–9]. To the best of our knowledge, there is limited empirical research confirming the signaling function of feedback in this context.

The feedback format has a critical impact on the contribution level in the repeated PGG setting. However, previous literature has manipulated the information conveyed in the feedback to investigate its effects. For example, [10] provided information regarding one of the contributions made by others in addition to the standard repeated PGG. They found that when the maximum contribution in a given period was exogenously chosen as the additional feedback and the manner in which the information was selected (i.e., maximum, minimum, or random) was unknown to the subjects, the average contribution level increased. The experiment conducted by [6] compares feedback with null information and information on the sum of partners’ contributions. Their findings indicate that the initial contributions in both treatments are not statistically different, thus challenging the validity of the strategic play hypothesis (referred to as the signal function in this paper). However, varying the informative power of feedback may simultaneously impact both the informational and signaling functions. Therefore, it is difficult to assert that the signaling function does not exist; it is plausible that the signaling and informational functions cancel each other out.

To isolate the signaling function, we manipulated the feedback format while keeping its informative content constant. Specifically, we considered two treatments in a between-subject design. In both treatments, participants receive feedback after making their contribution decisions in each period. In the simplified-feedback (SF) treatment, participants receive information about the current period, including their own contribution, the total group contribution, and their own profit. In the detailed-feedback (DF) treatment, in addition to the simplified feedback, participants receive feedback that also includes information about their own keeping (i.e., the amount of tokens not contributed), the sum of their partners’ contributions, and the historical information of all concluded periods.

It is important to note that the additional information provided in the DF treatment is redundant. Since the endowment, which remains constant throughout each period of the experiment, can only be allocated in contributing in the public good and keeping in their own pocket, participants can determine “their own keeping” by subtracting “their own contribution” from “the endowment of the period.” Similarly, participants can ascertain “the sum of partners’ contributions” by subtracting “their own contribution” from “the total group contribution.” Finally, the historical information solely records previous feedback and does not provide any additional information. Therefore, in terms of the informational function, both treatments contain feedback that has the same level of informativeness, which should not affect participants’ beliefs about others’ contributions in the subsequent period and, consequently, their contribution decisions. On the other hand, regarding the signaling function, the provision of detailed feedback exposes the signal more explicitly by presenting redundant information and historical feedback, thereby increasing the motivation to contribute as a signal of willingness to contribute. As a result, we expect that the subjects in DF treatment will exhibit a higher average contribution level compared to those in the SF treatment.

The use of feedback information may vary across subjects. Numerous public goods experiments have shown that the propensity for voluntary contribution varies among subjects. The related pioneering work is done by [11], in which they integrate strategy method into one-shot PGG experiment to elicit subjects’ cooperative disposition. [12] collects data from 17 replication studies of [11] and calibrates the criteria used to identify types so that the result of different studies can be compared to each other. In the combined data set with over 7,000 observations from 18 studies, they find that the phenomenon of heterogeneous cooperative disposition is stable: conditional cooperation is the predominant pattern; free-riding is frequent. For more detailed survey on the experimental studies with regard to phenomenon of heterogeneous cooperative disposition, please see [3,13].

A significant amount of participants, referred to as conditional cooperators (CCs), are predisposed to contribute in the one-shot PGG only if their partners do so as well. The motivation of CCs can be linked to a range of factors, including reciprocity, conformity, inequality aversion, anchoring, and/or confusion, as elucidated by [14]. In the repeated PGG, individuals exhibiting reciprocity tendencies are likely to engage in cooperative responses upon receiving feedback about others’ contributions. Additionally, these individuals may employ feedback as a strategic tool to indicate a willingness to cooperate. This is typically manifested through their contributions to the public account, with an anticipation of reciprocal cooperation from others [15]. Alternatively, if the driving forces behind individual behavior are conformity or inequality aversion, feedback is utilized as a source of information about the actions of others. This information prompts individuals to either align their actions with their peers in the case of conformity or to engage in behavior aimed at reducing disparities in payoffs when motivated by inequality aversion. Lastly, the precise roles of anchoring and confusion in motivating conditional cooperation, which remain conflated in [14]’s experimental setup, are not well understood. However, it seems unlikely that in such scenarios, individuals would systematically use feedback as a signaling mechanism. To summarize, of the factors previously discussed, only those CCs driven by reciprocity might consider feedback as a signaling mechanism, potentially influencing their contribution decisions through additional redundant information. Nevertheless, experimental studies designed to differentiate these underlying motivations suggest a limited and statistically marginal influence of reciprocity [14,16,17]. Therefore, we hypothesize that there will be no significant difference in the average contribution levels of CCs across treatments.

The second-highest proportion of participants, referred to as free riders (FRs), are predisposed to never contribute in the one-shot PGG, regardless of others’ actions. In contrast to CCs, who are driven by social preferences, FRs are motivated by the conventional assumption in economic theory that they should maximize their own monetary payoff. However, in a repeated PGG, FRs recognize that the future contributions of CCs are influenced by the feedback from current average contribution. Consequently, they tend to make a positive contribution as a signal of their willingness to cooperate in subsequent rounds [9]. Nevertheless, this motivation diminishes as the game comes close to its end [7,8]. Therefore, the FRs mainly utilize the signaling aspect of feedback. Considering the magnification of the signal effect caused by the inclusion of supplementary redundant information and historical feedback, we expect a higher average contribution from FRs in the DF treatment. To empirically examine the heterogeneous behavior between CCs and FRs as previously argued, we employ a one-shot PGG with strategy method similar to [11] to elicit subjects’ cooperative disposition in the first stage of the experiment.

## Experiment design and procedures

Similar to [5], our experiment comprises of two stages. In the first stage, we measured the subjects’ propensity for voluntary cooperation in a one-shot PGG played by four players using the strategy method. In the second stage, we observed the subjects’ contribution choices in a 20-period PGG played by four players using the partners protocol. It is important to note that the subjects were randomly rematched into groups between stage 1 and stage 2. This was done to ensure that the subjects’ belief in stage 2 was not influenced by the feedback received in stage 1.

The fundamental decision-making scenario is a typical linear PGG. In each round of the PGG, all four participants received an endowment of 20 tokens. These tokens could be either voluntarily contributed to a public good, known as the “project account,” during the experiment or kept in their personal accounts. The quantity of tokens that subject *i* decided to contribute is denoted as *x*_*i*_. The payoff function for subject *i* can be expressed as follows:

$$\pi_{i}=\text{20}-x_{i}+\text{0.4}\sum_{j=\text{1}}^{\text{4}}x_{j}$$ (1)

To contribute to the public good, the marginal payoff is 0.4, while the marginal cost is −1. In a one-shot PGG, rational individuals seeking to maximize their own monetary payoff would tend to behave as free riders and contribute nothing to the public good. All subjects were presented with the fundamental decision scenario described above in the instructions. Following the explanation of the scenario by the experimenter, ten control questions were administered to the subjects in order to facilitate their comprehension. Please refer to S1 File for the instruction in the English version.

**Fig 1:**
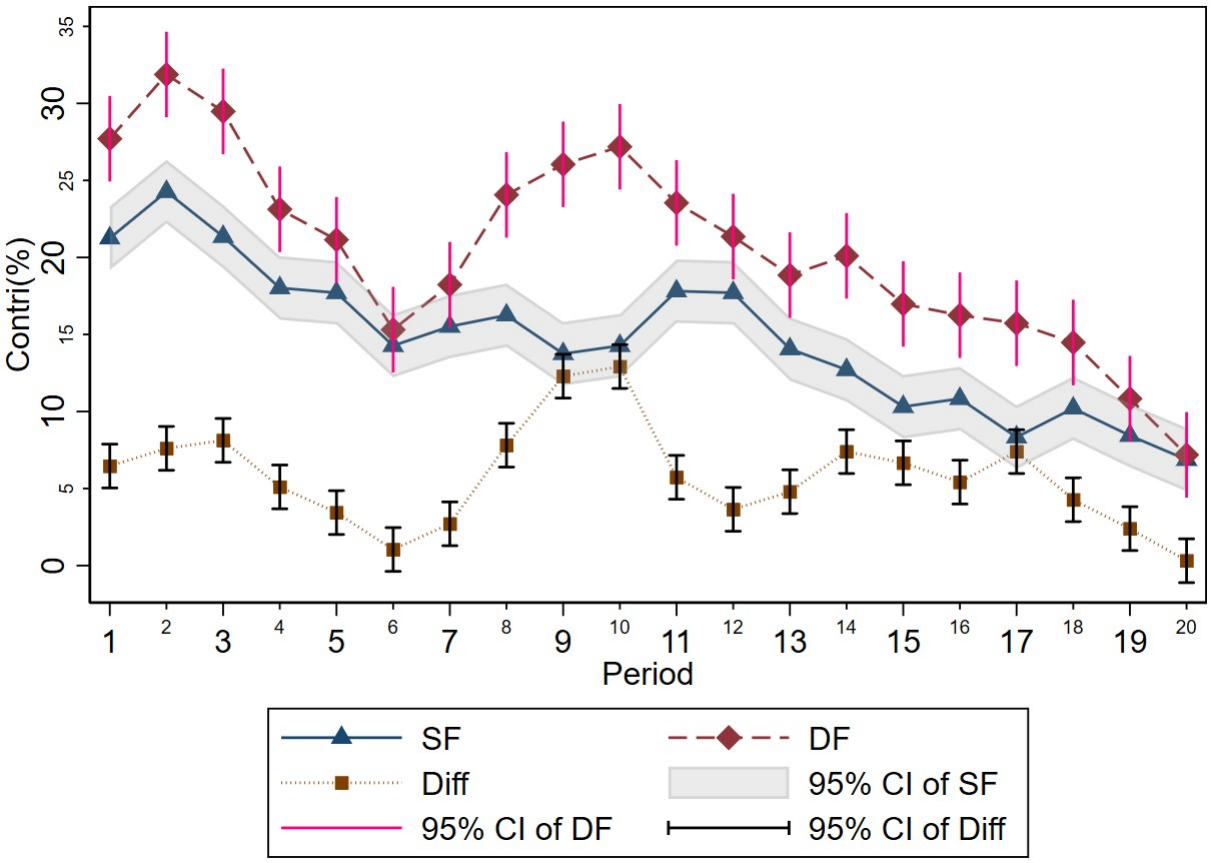
Average contributions over time for the SF treatment and DF treatment.

The decision situation in our experiment consisted of two stages. In the first stage, participants were asked to make two types of contribution decisions, namely, conditional and unconditional contributions. They were instructed to make both decisions privately, without any time constraints. In the conditional contribution task, participants were required to complete a contribution table. They were required to specify the amount they would be willing to contribute to the public good account corresponding to each of the 21 distinct average contribution levels of their fellow group members, ranging from 0 to 20 (check Fig 1 in the S1 File for the experimental screenshot). Once the conditional contribution decision was made, a new screen appeared prompting subjects to make an unconditional contribution decision. The unconditional contribution task simply asked how many of the 20 tokens the subject wished to invest into the public good. In order to ensure that every decision is thoroughly considered, the following contribution determination procedure ensures that all inputs within the contribution table, as well as unconditional contributions, have the potential to influence the payoffs for all subjects. Once decisions are finalized, a random selection mechanism identifies one subject from each group. For the other three group members, their unconditional contribution would be the relevant decision for their payoffs. The mean of these three unconditional contributions is calculated and then integrated into the contribution table of the randomly selected participant, thereby determining their specific contribution. Ultimately, the earnings for each participant are computed using Eq (1). In this first stage, we can ascertain the cooperative disposition of the subjects by referring to the contribution table.

After completing the first stage experiment, the subjects were randomly reassigned to new groups, and the second stage experiment began. During the second stage, subjects were required to make unconditional contributions for twenty rounds with the same group members. At the end of each round, the screen displayed feedback information that we attempted to manipulate. We consider two treatments in a between-subjects design: A simplified-feedback (SF) and a detailed-feedback (DF) treatment. In the SF treatment, subjects receive feedback that includes three components: (i) the number of tokens they invested in the public good account during the period, (ii) the total number of tokens invested by the group in the public good account during the period, and (iii) the number of tokens they earned in the period. In the DF treatment, in addition to the feedback provided in the SF treatment, subjects are given information on (iv) the number of tokens remaining in their private account, (v) the total contribution made by the other group members, and (vi) the historical information from all concluded periods. As mentioned previously, information (iv), (v), and (vi) are essentially redundant.

The experiment was conducted between December 7th and December 15th, 2019, at the Behavioral and Experimental Economic Research Laboratory (BEER Lab), located at Hubei University of Economics. The participants comprised adult undergraduate students representing diverse academic disciplines, excluding Economics, within the university. We exclude the students who majored in Economics because they might have learned the conventional theory of the public good game, which teaches that the profit maximization strategy is to free ride [18]. All participants provided written informed consent documents. Personal data that could identify participants were not collected. Research data from this experiment will be securely stored electronically within an encrypted folder and will be scheduled for deletion after an 8-year period (on December 7, 2027). The Scientific Review Committee at Hubei University of Economics conducted a thorough review and granted approval for this research. All authors confirm that the experiment adhered to the ethical regulations established by the Scientific Review Committee at Hubei University of Economics.

The experiment was designed and conducted using the experimental software “z-Tree,” developed by [19]. We conducted four sessions of the SF treatment and four sessions of the DF treatment, with 12 participants in each session. In total, 96 subjects were randomly assigned to either the SF treatment (48 subjects) or the DF treatment (48 subjects). None of the subjects participated in more than one session of the experiment. On average, each subject spent 1.5 hours and earned 42.2 RMB (about $5.99). Given the local hourly wage of 16 RMB at the time, we offered a monetary incentive that was 2.6 times the local wage. We believed this amount would sufficiently motivate the participants to approach the experiment seriously. At the time of the experiment, the exchange rate was 1 USD to 7.04 RMB, based on the data from the State Administration of Foreign Exchange of China.

## Experimental results

We begin by categorizing the subjects’ contribution table into three distinct categories, which represent the propensity for voluntary cooperation. Based on this categorization, we illustrate the distribution of subjects’ propensity for voluntary cooperation within our dataset. Subsequently, we provide empirical evidence for the signaling function of feedback in repeated PGG by comparing the average contributions of both the SF and DF treatments. Finally, we analyze the dynamics of contribution in PGG and report the distinct effects of redundant information on CC and FR.

### Propensity of voluntary cooperation

In the first stage of the experiment, we measured subjects’ propensity of voluntary cooperation, specifically their willingness to contribute given the average contribution of others. Similar to the classification in [11], we categorized all subjects into three distinct groups. To establish the categorization criteria, we define the contribution made by participant *i* in the contribution table as a vector $s^{i}=(s_{\text{0}}^{i},\;s_{\text{1}}^{i},\;\ldots ,\;s_{\text{20}}^{i})$. The definitions for the categories are outlined below.

- Conditional cooperator (CC): Participants are more likely to contribute when the average contribution of other partners is higher. Specifically, the Spearman’s rank correlation coefficient between *s*^*i*^ and (0, 1, …, 20) exhibits a statistically significant positive relationship at the 1% level.
- Free rider (FR): Participants’ contribution in the table are almost 0. Specifically, it is required that $\sum_{j=\text{0}}^{\text{20}}s_{j}^{i}/21<\text{1}$, and there is a statistically insignificant Spearman’s rank correlation coefficient between *s*^*i*^ and (0, 1, …, 20).
- Other types (OT): Participants whose contribution cannot be categorized as CC or FR.

Table 1 shows the distribution of cooperative dispositions among 96 participants across various treatments. In total, 51 subjects (53.13%) were categorized as CC, 20 subjects (20.83%) as FR, and 25 subjects (26.04%) fell into other types (OT). The p-values from both the Fisher’s exact test and Pearson’s chi-squared test do not provide sufficient evidence to reject the null hypothesis that there is no difference between the distributions. Given that the manipulation of the feedback format occurs during stage 2 of the experiment, this result confirms the homogeneity of the distribution of cooperative disposition among subjects in both groups.

**Table 1:** Distribution of cooperative disposition across treatments.

Treatment	CC		FR		OT	
	Frequency	%	Frequency	%	Frequency	%
SF	28	29.17	08	08.33	12	12.50
DF	23	23.96	12	12.50	13	13.54
Fisher’s exact test	[0.544]					
Pearson’s chi-squared test	1.33 [0.514]					

Note: P-values are reported in bracket.

### The effect of redundant information

In this section, we present the findings from the second stage of our experiment, which involved a 20-round repeated PGG employing the partner protocol. The contribution level is expressed as a percentage of the endowment. That is, Subject *i*’s contribution (in percentage) in period *t*, denoted as *x*_*it*_, is calculated as (*x*_*it*_/20) × 100%. Moreover, the higher the participants’ contributions to the public good account, the greater the level of cooperation within the group, leading to enhanced social benefits. Therefore, we interchangeably employ the terms “contribution” and “cooperation” in the subsequent discussion.

In the DF treatment, the average contribution of subjects across all 20 periods is 20.47%, markedly exceeding the 14.70% observed in the SF treatment. Statistical analysis reveals a p-value of 0.058 from the one-tailed t-test and a p-value of 0.059 from the two-tailed Mann-Whitney test, indicating a significant difference between the two treatments. This implies that providing redundant information in the DF treatment results in a statistically significant increase in average contributions. Furthermore, considering that the informative power remains consistent across both treatments, it suggests that the informational function of feedback does not have an impact. Therefore, this empirical finding provides evidence supporting the existence of a signaling function in the repeated PGG.

To further examine the signaling function of feedback in repeated PGG, we present the temporal patterns of contributions for the SF treatment, DF treatment, and the difference between the two in Fig 1. Compared to the SF treatment, the additional contributions in the DF treatment serve as a measure of the signaling effect resulting from the presence of redundant information. It is apparent that the average contributions in the DF treatment consistently surpass those in the SF treatment across periods, except for periods 6 and 20.

However, both SF treatment and DF treatment exhibit a decline in cooperation, which is consistent with findings from previous literature [3,20,21]. In the SF treatment, the average contribution decreases from 21.25% in the first period to 6.88% in the last period. In the DF treatment, the average contribution in the initial period is 27.71%, which decreases to 7.19% in the final period. Table 2 presents the empirical evidence of this trend through a primary regression analysis. In particular, we employ a gradual regression approach to examine the relationship between participants’ contributions and several variables: the period, a dummy variable denoting whether the participant received detailed feedback (DF = 1) or simplified feedback (DF = 0), and the interaction between the feedback format and the period. In columns (1) to (3), while controlling for the feedback format and its interaction term, we consistently find the estimated coefficients for the period are negative and statistically significant, indicating a decline in cooperation observed in both the SF treatment and the DF treatment. In columns (2) and (3), the coefficients of DF enter positively and significantly, suggesting that the redundant information contained in the DF treatment facilitates cooperation in groups, while controlling for the game period. Finally, the coefficient of the interaction term in column (3) does not significantly differ from zero, implying that the presence of redundant information does not affect the decline of cooperation in repeated PGG.

**Table 2:** Benchmark estimates.

	(1)		(2)		(3)	
period	-0.158***	(0.019)	-0.158***	(0.018)	-0.145***	(0.026)
DF			1.155***	(0.212)	1.434***	(0.441)
DF × period					-0.027	(0.037)
constant	5.175***	(0.222)	4.598***	(0.245)	4.458***	(0.312)
observations	1920		1920		1920	

Note: Standard errors are in parentheses.

*** indicate significance at 1%.

### Contribution dynamic and heterogeneous behavior

Extensive evidence from previous literature and our dataset documents the heterogeneous propensity for voluntary contributions. Consequently, it is natural to infer that the redundant feedback information affects participants with different cooperative dispositions in varying manners. Our analysis not only focuses on the full sample of the experiment but also analyzes the dynamics of contribution decisions made by individuals classified as CC and FR, respectively. To quantify the impact of redundant information on individual contribution choices in repeated PGG, we estimate the following equation, which captures the panel data dynamics for contributions [6]:

$${Contri}_{it}=\beta_{\text{0}}+\beta_{\text{1}}∙{Contri}_{it-\text{1}}+\beta_{\text{2}}∙{OtherContri}_{it-\text{1}}+\beta_{\text{3}}∙{DF}_{i}+\eta_{i}+\varepsilon_{it}$$ (2)

where each subject is indicated by index *i* and each period by index *t*. The equation explains the subjects’ contribution (*Contri*_*it*_) as a function of their own past contributions (*Contri*_*it*−1_), the lagged contribution of the other three group members (*OtherContri*_*it*−1_), a dummy variable representing the presence of redundant information in the feedback (*DF*_*i*_), and the subject-specific fixed effect (*η*_*i*_). The model is estimated using the generalized method of moments (GMM) to ensure parameter estimate consistency within the corresponding dynamic panel data structures. Specifically, we employed the system estimator developed by [22]. The result from the full sample of the experiment is summarized in column (1) of Table 3. In the column (2) and (3), we show the results from CCs and FRs, respectively. In addition, we report the results of two statistical tests, the Hansen J test and the second-order serial correlation test of differenced residuals (AR(2) test), proposed by [23] in Table 3. In all regressions, the statistics of the Hansen J test and AR(2) test are statistically insignificant, indicating that the instruments used are valid and the residuals are serially uncorrelated.

**Table 3:** Dynamic panel data regression.

	(1)		(2)		(3)	
	All		CC		FR	
*Contri*_*it*−1_	0.630***	(0.022)	0.540***	(0.046)	0.386***	(0.074)
*OtherContri*_*it*−1_	0.065***	(0.007)	0.136***	(0.036)	0.030	(0.028)
DF	0.159**	(0.067)	0.078	(0.205)	0.325*	(0.186)
constant	0.271***	(0.066)	0.030	(0.229)	0.705**	(0.258)
observations	1824		969		380	
subjects	96		51		20	
instruments	60		28		22	
Hansen J	64.760		24.271		17.977	
AR(2)	1.625		0.821		1.538	

Note: Standard errors are in parentheses.

*** indicates significance at 1%.

** indicates significance at 5%.

* indicates significance at 10%.

As shown in the first column of Table 3, the coefficient of *Contri*_*it*−1_ is positive and statistically significant at the 1% level, suggesting the existence of persistence in subjects’ contribution behavior over time. Furthermore, this finding remains consistent in column (2) and column (3). Given that individuals’ contribution decisions in the repeated PGG are highly dependent on their beliefs regarding other members’ contributions, they learn these beliefs from the feedback information. However, [6] found that the learning process is incomplete, that is, participants do not fully adapt their beliefs to the actual contributions of others. As a result, their contribution decisions exhibit persistence.

The estimate on *OtherContri*_*it*−1_ captures participants’ conditional cooperative behavior. As expected, the coefficient of *OtherContri*_*it*−1_ in column (2) is positive and strongly significant, indicating that a higher contribution by other members in the previous period leads to a higher contribution by CCs in the current period. The behavior of the CCs in repeated PGG is consistent with their cooperative preferences observed in the one-shot PGG. On the other hand, the coefficient of *OtherContri*_*it*−1_ in column (3) is positive but not significantly different from 0, indicating that the contribution decision of FR is irrelevant to other members’ contributions, even though it differs from the predisposition of never contributing in the one-shot PGG.

The positive contribution of FRs in the repeated PGG is motivated by the strategic signaling [9]. When comparing the SF and DF treatments in the experiment, the redundant information contained in detailed feedback provides no additional information about other members’ contributions. However, it exposes the signal more explicitly by presenting similar information and historical feedback. Hence, given that the motivation to signal is promoted in the DF treatment, FRs’ contribution should positively depend on DF. In Table 3, column (3) provides evidence supporting the hypothesis, as the coefficient of DF is significantly positive. Nevertheless, in column (2), the coefficient of DF is statistically insignificant, indicating that CCs do not utilize the signaling function of the feedback. In the dynamic panel analysis, it’s assumed that participants’ contributions are influenced by the contributions of other group members from the last period. This assumption is somewhat intuitive for the SF treatment. However, in the DF treatment, where historical information is provided in the feedback, we conducted additional regression analysis similar to Eq (2), incorporating a variable for the contributions of other group members from two periods prior (*OtherContri*_*it*−2_). The results, summarized in S4 Table 2 in S1 Table, reveal that the coefficient for *OtherContri*_*it*−2_ is not significantly different from zero, indicating that participants did not use information from periods before the last one in their decision-making process for contributions. Moreover, the main findings presented in Table 3 remain consistent in S4 Table 2 in S1 Table, indicating that the outcomes of our dynamic panel analysis demonstrate considerable robustness.

## Summary

The feedback contains information regarding others’ contribution behavior in the repeated PGG, which crucially affects participants’ beliefs and their subsequent contribution decisions. Theoretically, the information included in the feedback serves two purposes. Firstly, it can be used to acquire information about other members’ behavior. Secondly, it can be used to signal the willingness to cooperate to other members. In this paper, we experimentally investigate the sole impact of the signaling function of feedback on participants’ contributions in a repeated PGG.

In order to achieve the objective, it is crucial for the experimental design to effectively disentangle the impact of the signaling function from that of the informative function. We considered two treatments in a between-subjects design. The feedback in the SF treatment contains regular information that is commonly used in the literature of repeated PGG experiments. The feedback in DF treatment additionally contains more, but redundant, information. We argue that the additional information provided in the DF treatment is redundant because participants in the SF treatment can easily count and record such information. Therefore, the information contained in both treatments is exactly the same, ensuring that the informative function of the feedback does not affect participants’ contribution decisions.

The data from our repeated PGG experiment demonstrates that the average contribution of subjects in the DF treatment is significantly higher than that in the SF treatment. Given that the inclusion of redundant information in the DF treatment provides a more explicit signal exposure. Hence, the findings suggest that an increase in the signal’s effectiveness leads to a corresponding rise in participants’ inclination to make contributions, thereby signaling their cooperative intent. While the additional feedback provided in the DF treatment does not contribute new information, it may nonetheless affect participants’ anchoring biases or levels of confusion, thereby impacting their propensity for cooperative behavior. Future research endeavors should incorporate experiments specifically tailored to isolate and examine these residual factors.

Our results offer insights into various real-world situations where repeat interactions and feedback mechanisms are prevalent. For example, the reduction of carbon emissions, a complex public good issue that necessitates collective efforts from countries worldwide through international cooperation. Our findings emphasize the critical function of platforms where various entities can communicate and exhibit their commitment to this specific field, namely signaling their willingness to cooperate. Furthermore, even when multiple platforms, such as the United Nations Framework Convention on Climate Change Conference of the Parties (COP), Major Economies Forum on Energy and Climate (MEF), APEC Energy Ministers Meeting, and European Union Energy Ministers Meeting, provide repetitive and redundant information, our findings suggest their utility in enhancing global cooperation. However, given the non-linear nature of carbon emission scenarios, the Threshold Public Goods Game is a more appropriate model for the issues previously delineated. Consequently, an extensive examination of the feedback’s signaling function in the Threshold PGG is imperative.

Additionally, we consider the heterogeneous utilization of feedback among subjects in the paper. Specifically, we identify the significant types of cooperative dispositions, namely, CC (Conditional Cooperator) and FR (Free Rider), through a one-shot PGG employing a strategy method. The analysis of contribution dynamic in the repeated PGG shows that CCs’ contribution depends positively on the contributions of other members, but are not affected by redundant information. On the other hand, the contributions of FRs are irrelevant to the contributions of other members, but increase with the provision of redundant information. The findings present empirical evidence indicating that CCs employ feedback primarily for its informative function, whereas FRs utilize feedback primarily for its signaling function.

The theoretical contribution of this paper is to provide empirical evidence that feedback can serve as a signal in repeated PGG. Moreover, we identify the heterogeneous utilize of feedback across conditional cooperators and free riders. In practice, firms, communities, charities, and crowdfunding organizations provide feedback to facilitate contributions [10]. Based on our evidence, providing detailed but redundant information is helpful in promoting cooperation within organizations. Moreover, the provision of redundant information might complement other cooperation-facilitating mechanisms, such as punishment, which has been widely discussed in the literature. When punishment opportunities exist in a repeated PGG environment, individuals are more motivated to signal their willingness to cooperate. Consequently, we expect the promoting effect of redundant information on contributions to be greater. However, this conjecture requires further empirical evidence to verify. The findings of our paper illustrate that the use of feedback information differs among individuals based on their cooperative disposition. However, cooperative disposition itself can be intricately influenced by social preferences and personality traits. Therefore, it merits further exploration to understand how individual differences shape the use of feedback information. A further area for exploration involves examining the interdependent dynamics of human cooperation and the natural environment, where human activities both induce and respond to changes in environmental conditions. In such a repeated collective-risk social dilemma game, the feedback loop between individual behavior and environmental risks is fundamental to maintaining cooperation and responsible resource utilization [24]. Understanding how feedback among individuals (i.e., group outcomes in each period) and from the environment interrelate is crucial for more realistically tackling issues like climate change. These queries raised above are indeed worthy of further investigation.

## Supporting information

S1 File Experimental instruction for DF treatment. (DOCX)

S1 Data (CSV)

S2 Data (TXT)

S1 Table (DOCX)
