# Supplementary material for: The signaling role of feedback in the repeated public goods game: Experimental evidence from the laboratory
Source: PLoS One. 2024 Feb 29;19(2):e0299196. doi: 10.1371/journal.pone.0299196 (PMC10903900; doi:10.1371/journal.pone.0299196)
Supplement: S1 File — (DOCX) [file pone.0299196.s001.docx]

**S1 File. Experimental instruction for DF treatment.**

The experiment you are currently participating in is supported by several funding aimed at promoting academic research. By participating in this experiment, you can receive two types of profits, which will be paid in Chinese Yuan (RMB).

The earnings you obtain from participating in the experiment today can be divided into two parts:

The first part of the experiment involves an entry fee of 5 RMB. As long as you attend the experiment today, you will receive this fee.

In the second part, the outcomes will vary based on the decisions you make during the experiment. Your earnings will be determined by the number of tokens you accumulate throughout the experiment. The exchange rate is 1:0.08, which means that 1 experiment token is equivalent to 0.08 RMB. The highest possible earnings can reach 74 RMB, while the lowest possible earnings can be 13 RMB. Therefore, it is crucial to pay close attention to the following guidelines.

To ensure the experiment’s execution, the use of personal electronic devices is strictly prohibited. In order to prevent the use of mobile phones, we will temporarily hold and secure your phone until the end of the experiment when we distribute your final earnings for today’s participation. During the experiment, please refrain from engaging in conversations with others and avoid looking at other participants’ computer screens. The guide provided to you is confidential and strictly prohibits any form of communication with other participants during the experiment. At the conclusion of the experiment, kindly return this guide and refrain from removing it from the laboratory. If you have any questions, please raise your hand and patiently await the experimenter’s assistance. Please avoid direct questioning without the prompt from the experimenter. Non-compliance with these rules may lead to experiment interruption, resulting in the forfeiture of earnings related to today’s participation.

All the decisions you make today are confidential. Participants will be randomly assigned to groups of four individuals. Apart from the experimenters, nobody will know the composition of each group. We also assure you that no personal data about you will be disclosed.

After the experiment, we will ask you to fill out a questionnaire.

**1 Basic decisions**

We will provide a detailed explanation of the experimental procedures later. First, we will outline the process of making basic decisions, followed by a series of control questions designed to enhance your understanding.

In this experiment, you will be assigned as one of the four group members, each initially possessing 20 tokens for decision-making. These tokens can be allocated, either fully or partially, towards project investments. Any unused tokens designated for project investments will be automatically transferred to your personal account. The income generated will be derived from these two accounts. The following section provides a detailed explanation of the income distribution.

- **Income from private accounts**

Each token deposited into the private account earns one point. For instance, if you deposit twenty tokens into the private account (indicating zero investment in the project), you will accumulate 20 points from the private account. Depositing 6 tokens into the private account will result in an income of 6 points from the private account. It is important to note that other individuals, apart from yourself, will not receive any income from your private account.

- **Income from the project**

All members in the group receive equal rewards from your investments in the project. Likewise, you can also receive rewards from the tokens invested in the project by other members of the group. The income that each group member receives from the project is determined by the following formula:

$$\text{Income from the project}=\text{Total investment in the project}\times\text{0.4}$$

For example, if the total investment in the project by members of the group is 60 tokens, you and other group members would each receive an income of $\text{60}\times\text{0.4}=\text{24}$ points from the project. If the total contribution from all members in the group is 10 tokens, you and all other group members would each receive a reward of $\text{10}\times\text{0.4}=\text{4}$ points from the project.

- **Total income**

Your total income is the sum of income from private account and income from projects.

Income from private account ($=\text{20}-\text{project investment}$) $+$ Income from the project ($=\text{0.4}\times\text{total group project investment}$) $=$ Total income.

**2 Control questions**

Please answer the following control questions. The purpose of these questions is to familiarize you with the income calculation of these 20 tokens under different allocation decisions.

(1) Each group member is free to allocate 20 tokens as they wish. Assume that no group member (including yourself) invests in the project.

(A) What is your total income?

(B) What is the total income of the other group members?

(2) Each group member is free to allocate 20 tokens. Assume you invest 20 tokens in the project, and the other group members also invest 20 tokens in the project.

(A) What is your total income?

(B) What is the total income of the other group members?

(3) Each group member is free to allocate 20 tokens. Assume the other three members of the group have collectively invested 30 tokens in the project.

(A) If you invest 0 tokens in the project, what is your total income?

(B) If you invest 8 tokens in the project, what is your total income?

(C) If you invest 15 tokens in the project, what is your total income?

(4) Each group member is free to allocate 20 tokens. Assume you have invested 8 tokens in the project.

(A) The total investment by other members in the group towards the project is 7 tokens. What is your total income?

(B) The total investment by other members in the group towards the project is 12 tokens. What is your total income?

(C) The total investment by other members in the group towards the project is 22 tokens. What is your total income?

**3 Experimental procedure**

The experiment will utilize the basic decisions introduced earlier. The experiment consists of two parts, and in the end, you will receive a monetary reward based on your decisions (as well as the decisions of your group members). The first part of the experiment will only be conducted once, while the second part will be repeated 20 times. At the beginning of the first part of the experiment, all participants will be randomly assigned to a group of four individuals. Except for the experimenters, no one knows who is in which group, which means that the people around you may not necessarily be your group members.

- **Part 1**

As you are aware, you will be provided with 20 tokens that you can use to invest in projects or keep in your private account. In this part of the experiment, each participant is required to make two decisions. We will refer to these decisions as “conditional investment” and “unconditional investment.”

In the decision-making process for conditional investment, you are required to complete the “Investment table.” In the table, you need to indicate the number of tokens you want to invest in the project based on all the possible average investment of other group members (rounded to the nearest integer). This means that you can determine your own investment amount based on the average investment of other group members. You will understand immediately by referring to the following screen, as shown in Figure 1.

| 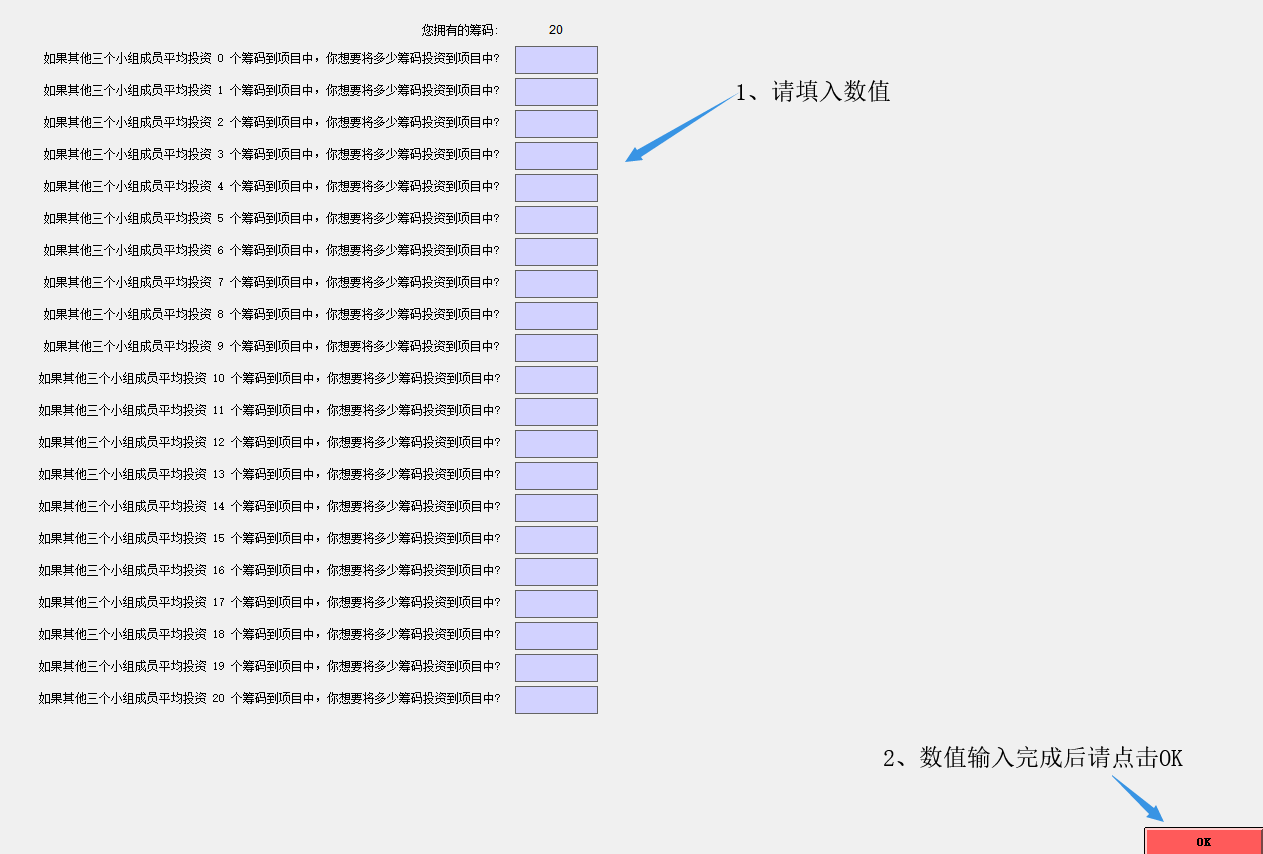 |
| --- |
| **Figure 1. Investment table** |

The numbers on the left side of the input boxes represent the average investments by other members of the group in the project. You need to enter the amount of tokens you are willing to invest in the project under these conditions. Please fill in all the input boxes. For example, if other group members on average invest 0 tokens in the project account, you need to specify how many tokens you want to invest in the project. If other group members on average invest 1, 2, or 3, and so on, tokens in the project, you can indicate how many tokens you are willing to invest. In each input box, you can enter an integer from 0 to 20. If you have filled in all the input boxes, please press the “OK” button.

Your second task is to make an unconditional investment, where you will decide how many out of the 20 tokens to invest in the project. You need to enter this quantity into the computer screen provided in Figure 2 below.

| 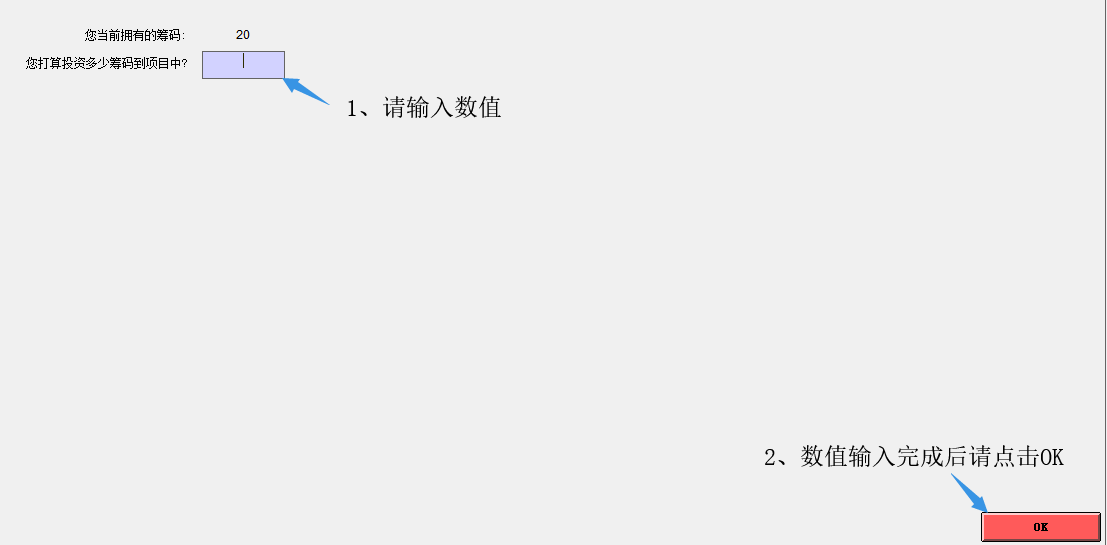 |
| --- |
| **Figure 2. Unconditional investment** |

- **Reward determination and random mechanisms**

After all participants in the experiment have completed the investment table and made their unconditional investment decisions, one member will be selected from each group through a random mechanism. The payoff for the randomly selected member will be related to the investment table. The payoffs for the other three members who were not randomly selected will be related to the unconditional investment. When filling out the investment table and making your unconditional investment decision, you are unaware of whether you will be selected through the random mechanism. Hence, careful consideration is necessary for both of these investment decisions, as they can potentially impact your overall payoff. Here are two examples:

Example 1: Assuming you have been selected by a random mechanism, it means that your payoff will be determined based on the investment table you completed earlier. The payoffs of the other three members in your group, on the other hand, are determined by the decision of unconditional investment. Let’s assume their unconditional investments are 0, 2, and 4 tokens, respectively. If, in the investment table, you specified an investment of 1 token when the average investment of other group members is 2 tokens, the total investment in the project would be $\text{0}+\text{2}+\text{4}+\text{1}=\text{7}$ tokens. Therefore, each member of the group can earn income from the project account, which is $\text{0.4}\times\text{7}=\text{2.8}$ points, in addition to the points in their respective private accounts. If, in the investment table, you specified an investment of 19 tokens when the average investment of other group members is 2 tokens, the total investment in the project would be $\text{0}+\text{2}+\text{4}+\text{19}=\text{25}$ tokens. Therefore, each member of the group can earn income from the project account, which is $\text{0.4}\times\text{25}=\text{10}$ points, in addition to the points in their respective private accounts.

Example 2: Assuming you have not been selected by the random mechanism, this indicates that your payoffs are determined by the decision of unconditional investment. Let’s assume your unconditional investment is 16 tokens, and the unconditional investments of the other two group members are 18 and 20 tokens, respectively. Therefore, the average unconditional investment among you and the other two group members is 18 tokens. If the selected group member specified in the previously completed investment table decides to invest 1 token in the project when the average investment of the other group members is 18 tokens, the total investment by the group in the project would be $\text{16}+\text{18}+\text{20}+\text{1}=\text{55}$ tokens. Consequently, each group member can earn $\text{0.4}\times\text{55}=\text{22}$ points from the project account, in addition to the points in their respective private accounts. If the selected member specified in the previously completed investment table decides to invest 19 tokens in the project when the average investment of the other group members is 18 tokens, the total investment by the group in the project would be $\text{16}+\text{18}+\text{20}+\text{19}=\text{73}$ tokens. Consequently, each group member can earn $\text{0.4}\times\text{73}=\text{29.2}$ points from the project account, in addition to the points in their respective private accounts.

- **Procedure of the random mechanism**

After all participants have completed the investment table and made their unconditional investment decisions, each group member will be assigned a number ranging from 1 to 4. Subsequently, a fair four-sided die will be rolled by the experimenter to randomly select a group member. If the die roll matches your assigned number, your payoff will be determined based on the previously filled investment table, while the payoffs of other group members will be determined based on their unconditional investment decisions. If the die roll matches another group member’s assigned number, your payoff will be determined based on the unconditional investment decision.

After each participant has been informed whether they have been selected or not, please enter the result in the displayed screen below, as illustrated in Figure 3. If you have been selected by the random mechanism, please enter 1; otherwise, please enter 0. Once the experimenter has confirmed, please press the “OK” button, and then you will be directed to the next page.

| 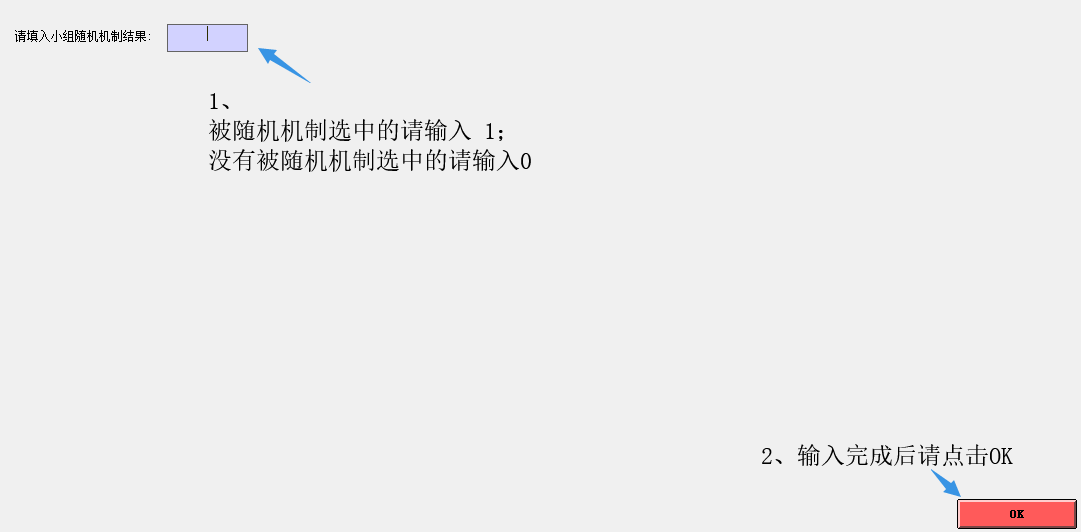 |
| --- |
| **Figure 3. Random mechanism** |

| 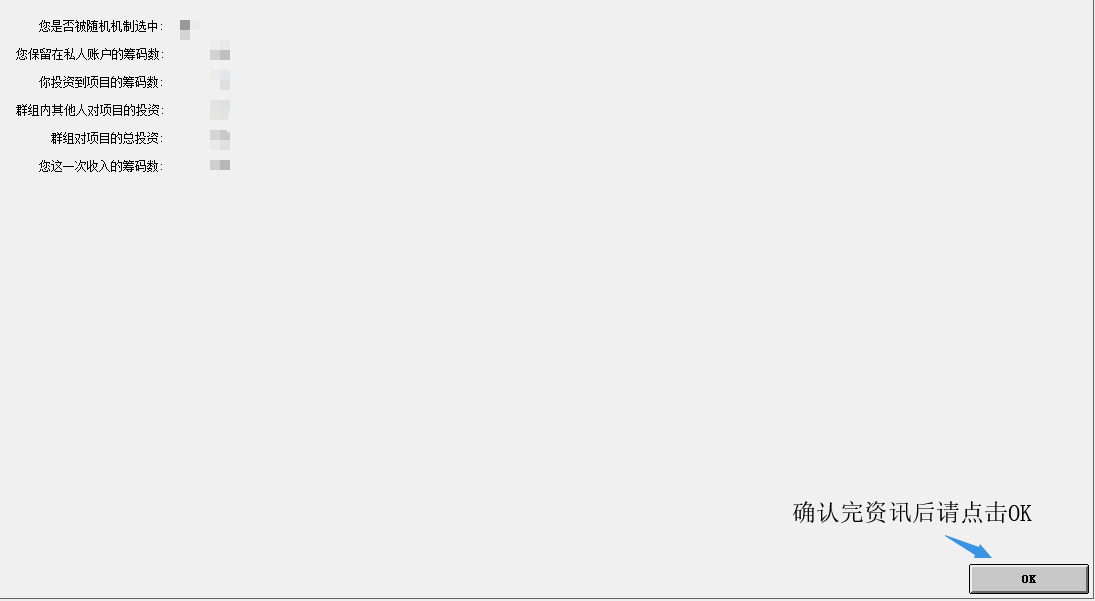 |
| --- |
| **Figure 4. Result of part 1** |

On the screen, as depicted in the Figure 4, you will see several pieces of information listed from top to bottom:

(1) Whether you have been selected by a random mechanism: If selected, it will be displayed as 1; if not, it will be displayed as 0.

(2) The number of tokens you have retained in your private account: This refers to the tokens that you have not invested in the project.

(3) The number of tokens you have invested in the project: This represents your investment decision.

(4) The total investment in the project by other individuals in the group: This indicates the total number of tokens invested by other members of the group.

(5) The total investment in the project by the group: This refers to the sum of the investments made by other individuals in the group, as well as your own investment.

(6) The number of tokens you will receive as income in this period: It is equal to the number of tokens you have in your private account plus the total investment by the group in the project multiplied by 0.4.

At this point, the first part of the experiment concludes. Please wait patiently for the experimenter to initiate the second part of the experiment.

- **Part 2**

This stage of the experiment consists of 20 rounds, and in each round, there are 20 tokens available for allocation. You are required to decide whether to invest the tokens in a project or place them in your private account.

- In the decision-making process of project investment, you are required to determine how many out of 20 tokens to invest in the project. Please enter this quantity into the computer screen following the interface shown in Figure 5.
- After determining your investment, please press the “OK” button.

| 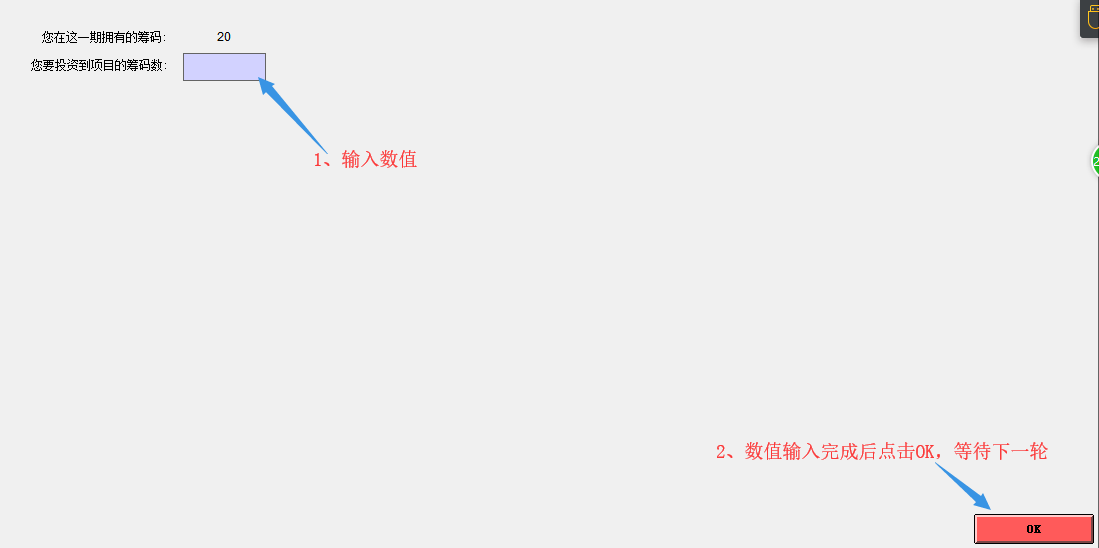 |
| --- |
| **Figure 5. Unconditional investment of part 2** |

| 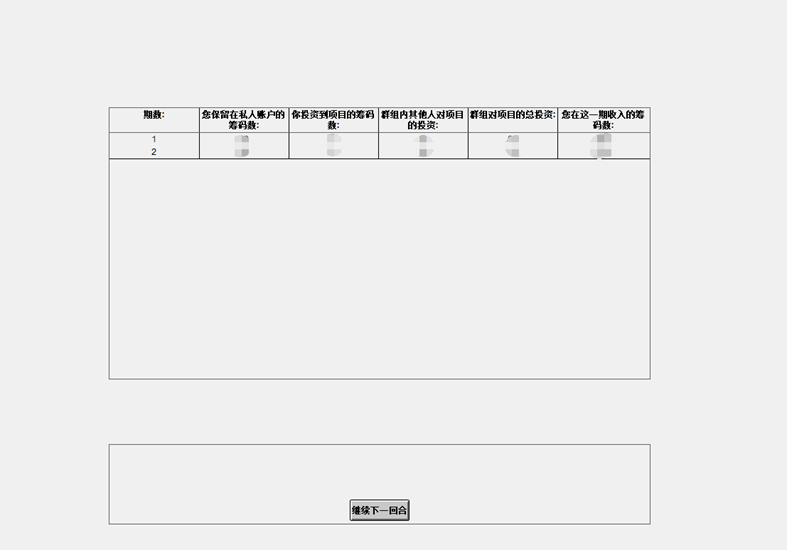 |
| --- |
| **Figure 6. Result of part 2** |

- As seen in the Figure 6, the screen displays the total investment amount for this period, as well as the points you have earned in this period. In addition to the above, all current and past historical information is shown on the computer screen.

On the screen, you will see several pieces of information from left to right:

(1) Number of periods: There will be a total of 20 rounds.

(2) The number of tokens you keep in your private account: This refers to the number of tokens that have not been invested in the project.

(3) The number of tokens you invest in the project: It refers to the investment decision you make.

(4) Other group members’ investments in the project: The total number of tokens invested by other members within the group.

(5) Group’s total investment in the project: The sum of others’ investments in the project plus your own investment.

(6) The number of tokens you will receive as income in this period: It is equal to the number of tokens you have in your private account plus the total investment by the group in the project multiplied by 0.4.

- When all participants in the experiment press the “Continue to Next Round” button, the experiment proceeds to the next round immediately.
- In each period, you will face the same situation and need to decide how many of the 20 tokens to invest in the project. Repeat this process until the end of the 20 periods.
- The points earned in each round will accumulate, and the total points earned in this part of the experiment will be the sum of points over 20 rounds.

At the end of the experiment, the experimenter will ask you to complete a questionnaire. Once you have finished filling out the questionnaire, please wait patiently as the experimenter will guide you to collect your mobile phone and today’s payment.

If you have any further questions, please raise your hand and wait for the experimenter to assist you.

**Copyright: S1 File. Experimental instruction for DF treatment. © 2023 by Chi-Hsiang Liu et al. is licensed under Attribution 4.0 International. To view a copy of this license, visit** [**http://creativecommons.org/licenses/by/4.0/**](http://creativecommons.org/licenses/by/4.0/)**.**
