# Supplementary material for: The signaling role of feedback in the repeated public goods game: Experimental evidence from the laboratory
Source: PLoS One. 2024 Feb 29;19(2):e0299196. doi: 10.1371/journal.pone.0299196 (PMC10903900; doi:10.1371/journal.pone.0299196)
Supplement: S1 Table — (DOCX) [file pone.0299196.s004.docx]

| **S4 Table 1. Summary statistics.** | | | | | | | | | | | |
| --- | --- | --- | --- | --- | --- | --- | --- | --- | --- | --- | --- |
|  | $Contri$ | | | | |  | $OtherContri$ | | | | |
| Period | Obs. | mean | std. dev | min | max |  | Obs. | mean | std. dev | min | max |
| Panel A: Detailed-feedback (DF) treatment | | | | | | | | | | | |
| 1 | 48 | 5.542 | 5.853 | 0 | 20 |  | 48 | 16.625 | 10.120 | 2 | 42 |
| 2 | 48 | 6.375 | 6.108 | 0 | 20 |  | 48 | 19.125 | 11.577 | 0 | 40 |
| 3 | 48 | 5.896 | 5.699 | 0 | 20 |  | 48 | 17.688 | 9.790 | 0 | 40 |
| 4 | 48 | 4.625 | 4.971 | 0 | 20 |  | 48 | 13.875 | 9.416 | 0 | 31 |
| 5 | 48 | 4.229 | 4.848 | 0 | 20 |  | 48 | 12.688 | 9.196 | 0 | 36 |
| 6 | 48 | 3.063 | 4.412 | 0 | 20 |  | 48 | 9.188 | 9.010 | 0 | 35 |
| 7 | 48 | 3.646 | 4.866 | 0 | 20 |  | 48 | 10.938 | 8.104 | 0 | 29 |
| 8 | 48 | 4.813 | 5.362 | 0 | 20 |  | 48 | 14.438 | 8.158 | 0 | 30 |
| 9 | 48 | 5.208 | 5.434 | 0 | 20 |  | 48 | 15.625 | 10.113 | 2 | 32 |
| 10 | 48 | 5.438 | 5.585 | 0 | 20 |  | 48 | 16.313 | 11.616 | 0 | 40 |
| 11 | 48 | 4.708 | 5.299 | 0 | 20 |  | 48 | 14.125 | 11.488 | 0 | 36 |
| 12 | 48 | 4.271 | 5.342 | 0 | 20 |  | 48 | 12.813 | 11.238 | 0 | 36 |
| 13 | 48 | 3.771 | 4.777 | 0 | 20 |  | 48 | 11.313 | 9.621 | 0 | 32 |
| 14 | 48 | 4.021 | 4.871 | 0 | 20 |  | 48 | 12.063 | 9.368 | 0 | 34 |
| 15 | 48 | 3.396 | 4.784 | 0 | 20 |  | 48 | 10.188 | 8.562 | 0 | 32 |
| 16 | 48 | 3.250 | 4.888 | 0 | 20 |  | 48 | 9.750 | 9.901 | 0 | 32 |
| 17 | 48 | 3.146 | 4.864 | 0 | 20 |  | 48 | 9.438 | 8.186 | 0 | 28 |
| 18 | 48 | 2.896 | 4.530 | 0 | 20 |  | 48 | 8.688 | 9.103 | 0 | 27 |
| 19 | 48 | 2.167 | 4.304 | 0 | 20 |  | 48 | 6.500 | 8.241 | 0 | 28 |
| 20 | 48 | 1.438 | 4.217 | 0 | 20 |  | 48 | 4.313 | 7.438 | 0 | 25 |
| Panel B: Simplified-feedback (SF) treatment | | | | | | | | | | | |
| 1 | 48 | 4.250 | 4.493 | 0 | 17 |  | 48 | 12.750 | 8.461 | 0 | 32 |
| 2 | 48 | 4.854 | 4.981 | 0 | 20 |  | 48 | 14.563 | 10.244 | 2 | 35 |
| 3 | 48 | 4.271 | 4.685 | 0 | 20 |  | 48 | 12.813 | 11.064 | 1 | 40 |
| 4 | 48 | 3.604 | 3.988 | 0 | 15 |  | 48 | 10.813 | 9.387 | 0 | 38 |
| 5 | 48 | 3.542 | 4.222 | 0 | 17 |  | 48 | 10.625 | 9.812 | 0 | 31 |
| 6 | 48 | 2.854 | 4.110 | 0 | 17 |  | 48 | 8.563 | 9.983 | 0 | 30 |
| 7 | 48 | 3.104 | 4.651 | 0 | 20 |  | 48 | 9.313 | 11.145 | 0 | 37 |
| 8 | 48 | 3.250 | 4.393 | 0 | 17 |  | 48 | 9.750 | 10.412 | 0 | 37 |
| 9 | 48 | 2.750 | 3.710 | 0 | 17 |  | 48 | 8.250 | 8.504 | 0 | 28 |
| 10 | 48 | 2.854 | 4.278 | 0 | 20 |  | 48 | 8.563 | 8.965 | 0 | 30 |
| 11 | 48 | 3.563 | 5.442 | 0 | 20 |  | 48 | 10.688 | 11.441 | 0 | 41 |
| 12 | 48 | 3.542 | 5.132 | 0 | 20 |  | 48 | 10.625 | 9.655 | 0 | 36 |
| 13 | 48 | 2.813 | 3.841 | 0 | 15 |  | 48 | 8.438 | 8.279 | 0 | 31 |
| 14 | 48 | 2.542 | 3.673 | 0 | 15 |  | 48 | 7.625 | 8.160 | 0 | 35 |
| 15 | 48 | 2.063 | 3.448 | 0 | 17 |  | 48 | 6.188 | 7.956 | 0 | 29 |
| 16 | 48 | 2.167 | 4.274 | 0 | 20 |  | 48 | 6.500 | 8.430 | 0 | 28 |
| 17 | 48 | 1.667 | 3.218 | 0 | 16 |  | 48 | 5.000 | 6.284 | 0 | 19 |
| 18 | 48 | 2.042 | 3.608 | 0 | 15 |  | 48 | 6.125 | 8.414 | 0 | 31 |
| 19 | 48 | 1.688 | 3.428 | 0 | 15 |  | 48 | 5.063 | 7.851 | 0 | 27 |
| 20 | 48 | 1.375 | 3.343 | 0 | 17 |  | 48 | 4.125 | 7.561 | 0 | 33 |

| **S4 Table 2. Robustness check.** | | | | | | |
| --- | --- | --- | --- | --- | --- | --- |
|  | **(1)** | | **(2)** | | **(3)** | |
|  | **All** | | **CC** | | **FR** | |
| ${Contri}_{it-\text{1}}$ | 0.546*** | (0.018) | 0.634*** | (0.016) | 0.175*** | (0.007) |
| ${OtherContri}_{it-\text{1}}$ | 0.037*** | (0.012) | 0.110*** | (0.009) | 0.043 | (0.031) |
| ${OtherContri}_{it-\text{2}}$ | -0.011 | (0.012) | -0.006 | (0.006) | 0.005 | (0.025) |
| DF | 0.330** | (0.139) | 0.065 | (0.052) | 0.412* | (0.227) |
| constant | 0.575*** | (0.119) | 0.083 | (0.060) | 0.547** | (0.195) |
| observations | 1728 | | 918 | | 360 | |
| subjects | 96 | | 51 | | 20 | |
| instruments | 65 | | 46 | | 20 | |
| Hansen J | 52.494 | | 42.677 | | 12.901 | |
| AR(2) | 1.631 | | 0.845 | | 1.344 | |
| Note: Standard errors are in parentheses,  *** indicates significance at 1%,  ** indicates significance at 5%,  * indicates significance at 10%. | | | | | | |
